# Diagnostic performance of blood inflammatory markers for tuberculosis screening in people living with HIV

**DOI:** 10.1371/journal.pone.0206119

**Published:** 2018-10-23

**Authors:** Katherine Farr, Resmi Ravindran, Luke Strnad, Emily Chang, Lelia H. Chaisson, Christina Yoon, William Worodria, Alfred Andama, Irene Ayakaka, Priscilla Bbosa Nalwanga, Patrick Byanyima, Nelson Kalema, Sylvia Kaswabuli, Winceslaus Katagira, Kyomugisha Denise Aman, Emmanuel Musisi, Nuwagaba Wallen Tumwine, Ingvar Sanyu, Robert Ssebunya, J. Lucian Davis, Laurence Huang, Imran H. Khan, Adithya Cattamanchi

**Affiliations:** 1 Division of Pulmonary and Critical Care Medicine, University of California San Francisco and Zuckerberg San Francisco General Hospital, San Francisco, California, United States of America; 2 Curry International Tuberculosis Center, University of California San Francisco, San Francisco, California, United States of America; 3 Department of Pathology and Laboratory Medicine, University of California at Davis, Sacramento, California, United States of America; 4 Division of Infectious Diseases, Oregon Health & Science University, Portland, Oregon, United States of America; 5 Epidemiology Programs, Oregon Health & Science University-Portland State University School of Public Health, Portland, Oregon, United States of America; 6 Division of HIV, Infectious Diseases, and Global Medicine, Department of Medicine, University of California San Francisco and Zuckerberg San Francisco General Hospital, San Francisco, California, United States of America; 7 Department of Epidemiology, Johns Hopkins Bloomberg School of Public Health, Baltimore, Maryland, United States of America; 8 Department of Medicine, School of Medicine, Makerere University, Kampala, Uganda; 9 Division of Respiratory Medicine, Department of Medicine, Mulago Hospital, Kampala, Uganda; 10 Department of Medicine, Mulago Hospital, Makerere University, Kampala, Uganda; 11 Infectious Diseases Research Collaboration, Kampala, Uganda; 12 International Multidisciplinary Programme to Address Lung Health and TB in Africa, Liverpool School of Tropical Medicine, Liverpool, United Kingdom; 13 Infectious Diseases Institute, Department of Research, Makerere College Health Sciences, Makerere University, Kampala, Uganda; 14 Department of Biochemistry, College of Natural Sciences, Makerere University, Kampala, Uganda; 15 Department of Epidemiology of Microbial Diseases, Yale School of Public Health, New Haven, Connecticut, United States of America; 16 Section of Pulmonary, Critical Care, and Sleep Medicine, Yale School of Medicine, New Haven, Connecticut, United States of America; King's College London, UNITED KINGDOM

## Abstract

**Background:**

Approaches to screening for active tuberculosis (TB) among people living with HIV are inadequate, leading to missed diagnoses and poor implementation of preventive therapy.

**Methods:**

Consecutive HIV-infected adults hospitalized at Mulago Hospital (Kampala, Uganda) between June 2011 and July 2013 with a cough ≥ 2 weeks were enrolled. Patients underwent extensive evaluation for pulmonary TB. Concentrations of 43 cytokines/chemokines were measured at the same time point as C-reactive protein (CRP) in banked plasma samples using commercially-available multiplex kits. Advanced classification algorithms were used to rank cytokines/chemokines for their ability to identify TB, and to model the specificity of the top-ranked cytokines/chemokines individually and in combination with sensitivity constrained to ≥ 90% as recommended for TB screening.

**Results:**

The median plasma level of 5 biomarkers (IL-6, INF-γ, MIG, CRP, IL-18) was significantly different between patients with and without TB. With sensitivity constrained to 90%, all had low specificity with IL-6 showing the highest specificity (44%; 95% CI 37.4–49.5). Biomarker panels were found to be more valuable than any biomarker alone. A panel combining IFN-γ and IL-6 had the highest specificity (50%; 95% CI 46.7–53.3). Sensitivity remained high (>85%) for all panels among sputum smear-negative TB patients.

**Conclusions:**

Direct measurement of unstimulated plasma cytokines/chemokines in peripheral blood is a promising approach to TB screening. Cytokine/chemokine panels retained high sensitivity for smear-negative TB and achieved improved specificity compared to individual cytokines/chemokines. These markers should be further evaluated in outpatient settings where most TB screening occurs and where other illnesses associated with systematic inflammation are less common.

## Introduction

Tuberculosis (TB) now ranks as the most common infectious disease cause of death and accounts for approximately one third of all deaths in people living with HIV (PLHIV) [1, 2]. To combat this deadly co-infection, the World Health Organization (WHO) articulated the "3 I's" strategy for reducing the burden of TB among PLHIV [3]. Key parts of this strategy include intensified case finding (ICF) consisting of screening all PLHIV for active TB at every healthcare encounter, followed by isoniazid preventative therapy (IPT) for those who screen negative and diagnostic testing for those who screen positive [4, 5]. However, the uptake and efficiency of IPT and ICF are limited by the lack of a TB screening test that has high enough sensitivity to rule-out TB for patients who test negative—thereby facilitating IPT initiation—and moderate specificity to identify a smaller subset of patients who require confirmatory TB diagnostic testing, thereby facilitating ICF.

Currently, the WHO recommends TB screening be performed using a four-part symptom screen alone or in conjunction with chest radiography (CXR) for PLHIV in high burden settings [5, 6]. Although the symptom screen has high sensitivity, recent data indicate that nearly all PLHIV are symptom screen-positive, particularly in sub-Saharan Africa [6–14]. The addition of CXR improves specificity, but cost and infrastructure requirements make implementation difficult at many HIV/AIDS clinics [6, 11, 14–16]. Thus, TB screening currently results in few PLHIV being identified as candidates for IPT and the vast majority requiring confirmatory testing. For these reasons, a low-cost and accurate screening test that can be performed by front-line health workers has been ranked among the highest priority needs for TB diagnostics [17, 18].

Measurement of inflammatory markers, cytokines and chemokines holds promise for facilitating TB screening [19, 20]. Previous studies have analyzed the accuracy of individual inflammatory markers such as C-reactive protein (CRP) and cytokines/chemokines such as interferon gamma (INF-γ), interferon gamma-induced protein (IP-10), monokine induced by interferon-γ (MIG), interleukin 6 (IL-6), and interleukin 18 (IL-18) for diagnosing active TB [21–27]. Of these, CRP has shown the most promise as a potential TB screening tool with high (89%) sensitivity for active TB and variable specificity (generally higher among outpatients and lower among hospitalized patients) [10, 22, 28–31]. We hypothesized that a panel of inflammatory markers, cytokines and/or chemokines measured directly in blood samples would have higher diagnostic accuracy for TB than CRP alone. To test this hypothesis, we measured levels of CRP and 43 cytokines/chemokines in stored plasma samples from an inpatient cohort of HIV-infected adults with presumptive pulmonary TB.

## Methods

### Study population and setting

We analyzed stored plasma samples from consecutive HIV-infected adults enrolled between June 2011 and July 2013 in an ongoing study, the International HIV-associated Opportunistic Pneumonias (IHOP) Study, of patients admitted to Mulago Hospital in Kampala, Uganda with cough for ≥2 weeks. Mulago Hospital is the national referral hospital in Uganda. As part of the parent study, patients submitted two sputum samples for acid-fast bacilli (AFB) smear microscopy (LED fluorescence microscopy) and liquid mycobacterial culture (MGIT), and one sample for GeneXpert MTB/RIF testing [32, 33]. Patients were followed-up two months after enrollment to assess for clinical improvement and repeat microbiologic assessment for TB if symptoms had not improved. Blood samples were collected in EDTA anti-coagulant at the time of enrollment and plasma was stored at -80 degrees Celsius. For this study, we included consecutive HIV-infected adult patients who 1) did not have a prior history of TB; 2) had sufficient sputum culture results to assess the reference standard (see Outcome Definition below); and 3) had a stored plasma sample available for analysis. The parent study (IHOP) was approved by institutional review boards at the University of California San Francisco and Makerere University, and by the Uganda National Council for Science and Technology. Informed written consent was obtained for participants at the time of their original enrollment as part of the IHOP studies. As this study represents a secondary analysis from that cohort, additional informed consent was not required. All data and samples were obtained and stored according to protocols approved by the Committee on Human Research at the University of California San Francisco, the School of Medicine Research Ethics Committee at Makerere University (Kampala, Uganda), and the Uganda National Council for Science and Technology.

### Multiplex cytokine/chemokine analysis

Two separate multiplex panels were used to measure a total of 43 cytokines/chemokines in plasma samples from 74 patients that had undergone only a single freeze-thaw cycle to minimize degradation. Samples were stored for approximately three years at -70 C prior to analysis. One kit included 41 cytokines/chemokines (EMD Millipore, Billerica, MA): Soluble CD40 ligand (sCD40L), vascular endothelial growth factor (VEGF), tumor necrosis factor (TNF)-α, TNF-β, transforming growth factor (TGF)-α, chemokine ligand 5/RANTES (CCL5), platelet-derived growth factor (PDGF)-AB/BB, PDGF-AA, macrophage inflammatory protein (MIP)-1β, MIP-1α, macrophage-derived chemokine (MDC), monocyte chemotactic protein (MCP)-1, MCP-3, interleukin (IL)-17, IL-15, IL-13, IL-12 (p70), IL-12 (p40), IL-10, IL-9, IL-8, IL-7, IL-6, IL-5, IL-4, IL-3, IL-2, IL-1ra, IL-1β, IL-1α, IFN-γ, IFN-α2, IP-10, growth-related oncogene (GRO), granulocyte macrophage colony-stimulating factor (GM-CSF), granulocyte-colony stimulating factor (G-CSF), fractalkine, Fms-related tyrosine kinase 3 (Flt-3) ligand, fibroblast growth factor (FGF)-2, eotaxin, and epidermal growth factor (EGF). The second kit (Bio-Rad, Hercules, CA) included 2 cytokines/chemokines: monokine induced by interferon-γ (MIG) and IL-18 [21]. Based on initial results (See Results: Biomarker Selection), 6 cytokines/chemokines were selected for further evaluation in 262 patients using custom-ordered multiplex kits: IFN-γ, IL-6, GRO, MDC (EMD Millipore, Billerica, MA), and IL-18 and MIG (Bio-Rad, Hercules, CA). The multiplex analysis was performed in duplicate on all samples according to the manufacturer’s recommendations at the Center for Comparative Medicine Laboratory, University of California, Davis. In addition to cytokines/chemokines levels (pg/ml), CRP levels (mg/L) were measured on a second aliquot of plasma in the Zuckerberg San Francisco General Hospital (ZSFG) Chemistry Laboratory by a turbidimetric method using a Beckman Coulter Analyzer. Technicians measuring cytokine/chemokine levels and CRP levels were blinded to clinical data including TB status. Patients were excluded from the data analysis if there was insufficient plasma to perform CRP measurement, results of duplicate biomarker measurements were not available, or the coefficient of variation was >0.3 between duplicate measurements.

### Outcome definition

We classified patients as having TB if sputum mycobacterial culture results were positive and speciation testing confirmed *Mycobacterium tuberculosis* complex, or if culture results were negative but both AFB smear and GeneXpert MTB/RIF results were positive. We classified patients as not having TB if all microbiologic testing for TB–including at least two liquid culture results–was negative and patients either had clinical improvement without anti-TB therapy or an alternate diagnosis established by the 2-month follow-up visit.

### Data analysis

We compared the differences in median biomarker responses between TB and non-TB patients using the Mann-Whitney test. We performed random forest modeling using two-thirds of the dataset to rank the relative variable importance of each biomarker in differentiating between TB and non-TB patients based on a mean decrease in Gini impurity index, a measure of heterogeneity in a group. A greater decrease in Gini impurity index indicates better classification, thus indicating a biomarker has greater relative importance [34, 35]. We subsequently built biomarker panels starting with the two top-ranked biomarkers based on variable importance and including the next highest ranked biomarker one at a time. For each panel, we developed a prediction algorithm for identifying TB using SuperLearner, a machine-learning ensemble that compares different learners (prediction methods) using cross-validated risk [36, 37]. SuperLearner builds a weighted linear combination of the included learners favoring those that minimize mean squared error to return predictions on the dataset using cross-validation to prevent over-fitting. For this analysis, we included logistic regression, Bayes’ generalized linear models, lasso, random forest, and a null model (in order to assess relative performance with other methods) as candidate learners [38–40]. After fitting the SuperLearner algorithm with two-thirds of the dataset, we applied it to the entire dataset and used the predicted probabilities of TB obtained after 10-fold cross-validation to calculate sensitivity, specificity, and cross-validated area under the curve (AUC), along with associated 95% confidence intervals (CI). For all biomarker panels, we constrained sensitivity to be 90% or higher based on the WHO’s target product profile for a TB screening test [7]. All analyses were performed using R, version 3.0.2.

## Results

**Fig 1:**
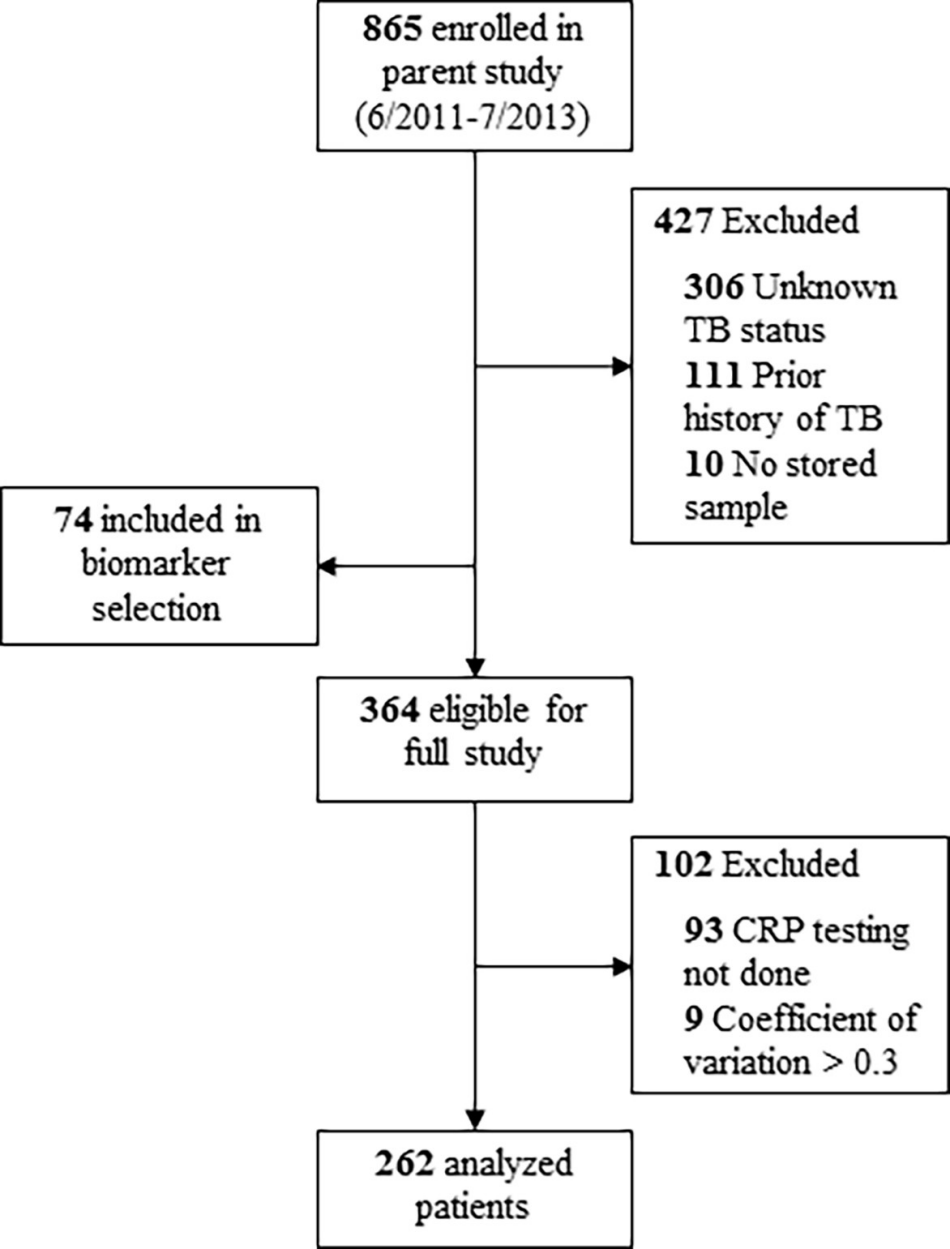
Patient selection. Of 865 patients enrolled in the parent study, 427 (49%) did not meet eligibility criteria, and 74 (9%) were included only for biomarker selection. Of the 364 patients eligible for the full study, 102 (28%) were excluded from the analysis. Abbreviations: TB, tuberculosis; CRP, C-reactive protein.

Of 865 patients enrolled in the parent study, 306 (35%) did not have a definite TB status, 111 (13%) had prior TB, and 10 (1%) had no stored sample. Data from the first 74 eligible patients (66% of whom were culture-positive for TB) were used for biomarker selection only. Of the remaining 364 patients, 9 (2%) showed a coefficient of variation >0.3 for at least one cytokine/chemokine, while 93 (25%) did not have CRP testing performed (Fig 1). Thus, 262 patients were included in the main analysis. Among the 262 patients, 150 (57%) were female, median age was 34 years (IQR 29–39), median CD4 count was 86 cells/μL (IQR 24–257), 65 (25%) were on anti-retroviral therapy, and 43% had advanced illness confining them to bed rest for at least part of each day. Of the included participants, 155 (59%) had culture-positive TB (68% smear-positive and 32% smear-negative) and 107 (41%) were assessed to be TB-negative based on microbiological evaluation and clinical follow-up. Of note, there were no significant differences in clinical or demographic characteristics among patients included and excluded from the analysis (data not shown).

### Biomarker selection (N = 74)

The median level (pg/ml) of 13 of 43 cytokines/chemokines measured was significantly different between patients with (n = 49) and without (n = 25) TB (S1 Table). The median level of CRP was also significantly higher in TB patients compared to patients without TB. Median levels were ≥2-fold higher among patients with TB than patients without TB for INF-γ, MIG, IL-6, IL-18, and G-CSF and were ≥1.5-fold higher for CRP, IL-8, IP-10, GRO, SCD-40L, TNF-α, and IL-1α. Results were similar when biomarkers were considered together and ranked by variable importance (S2 Table). The median level of 6 of the top 10 biomarkers based on variable importance (INF-γ, IL-18, CRP, IL-6, GRO, IP-10) was ≥1.5-fold higher among patients with TB than patients without TB. Two of the biomarkers (MDC and IL-17) had levels that were lower among patients with TB than patients without TB.

We selected the top 7 biomarkers (INF-γ, IL-18, CRP, IL-6, MDC, GRO, and IP-10) identified as promising based on variable importance for further analysis based on feasibility considerations. However, we replaced IP-10 with MIG for further analysis despite a lower variable importance score because of the ≥2-fold difference in the medial level of MIG between patients with and without TB, prior data demonstrating that MIG and IP-10 levels were highly correlated, and prior data suggesting that IP-10 is more likely to degrade after long-term storage [21, 41].

### Diagnostic accuracy (N = 262)

Among the remaining 262 patients, the median plasma level of 5 of the 7 selected biomarkers (IL-6, INF-γ, MIG, CRP, IL-18) was significantly different between patients with and without TB (Table 1). Median levels were ≥3-fold higher among TB patients for IL-6 and INF-γ, ≥2-fold higher for MIG and CRP, and 1.9-fold higher for IL-18. When considered together, the rank order from highest to lowest based on variable importance was IFN-γ, IL-6, MIG, IL-18, CRP, MDC, and GRO (Table 1).

**Table 1:** Variable importance and median concentrations (pg/ml) of 7 selected biomarkers (N = 262).

Biomarker	Variable Importance Ranking	No TB (n = 107)	TB (n = 155)	Fold Difference	p-value
		Median (IQR)	Median (IQR)		
IFN-γ	1	20 (11–34)	61 (36–126)	3.1	<0.001
IL-6	2	23 (13–50)	77 (40–130)	3.3	<0.001
MIG	3	2994 (1508–6425)	7741 (3346–13495)	2.6	<0.001
IL-18	4	248 (137–438)	482 (272–817)	1.9	<0.001
CRP (mg/l)	5	69 (23–158)	140 (78–209)	2.0	<0.001
MDC	6	788 (532–1169)	673 (518–925)	0.9	0.064
GRO	7	1390 (963–1931)	1432 (1111–1858)	1.0	0.738

Abbreviations: TB, tuberculosis; IQR, interquartile range, INF-γ, Interferon gamma; IL-6, Interleukin-6; MIG, Monokine induced by interferon-γ; IL-18, Interleukin-18; CRP, C-reactive protein; MDC, Macrophage-derived chemokine; GRO, Growth-related oncogene.

With sensitivity constrained to be at least 90%, all 7 individual biomarkers had low specificity for active TB (S3 Table). IL-6 had the highest specificity (44%, 95% CI 37.4–49.5). In the assessment of biomarker panels, with sensitivity constrained to 90%, specificity was higher with the combination of IFN-γ and IL-6 (50%, 95% (CI 46.7–53.3) than for either biomarker alone (Table 2). Adding the next top-ranked biomarker resulted in decreasing specificity, but the specificity of all panels containing from 2 to 7 of the top-ranked biomarkers was higher than that for the individual biomarkers included, except for IL-6. When applying TB risk cut-off points obtained from the full dataset, sensitivity remained high (>85%) for all biomarker panels among sputum smear-negative TB patients (Table 3).

**Table 2:** Accuracy of biomarker panels with sensitivity constrained to 90% (N = 262).

Biomarker Panel	Specificity (95% CI)	cvAUC
IFN-γ, IL-6	50.4 (46.7–53.3)	0.844
IFN-γ, IL-6, MIG	48.3 (44.9–51.4)	0.830
IFN-γ, IL-6, MIG, IL-18	46.6 (43.0–49.5)	0.851
IFN-γ, IL-6, MIG, IL-18, CRP	40.3 (36.4–43.0)	0.825
IFN-γ, IL-6, MIG, IL-18, CRP, GRO	41.8 (37.4–45.8)	0.826
IFN-γ, IL-6, MIG, IL-18, CRP, GRO, MDC	40.3 (36.4–43.9)	0.836

Abbreviations: TB, tuberculosis; CI, confidence interval; cvAUC, cross-validated area under the receiver operator curve; INF-γ, Interferon gamma; IL-6, Interleukin-6; MIG, Monokine induced by interferon-γ; IL-18, Interleukin-18; CRP, C-reactive protein; MDC, Macrophage-derived chemokine; GRO, Growth-related oncogene.

**Table 3:** Sensitivity of biomarker panels among sputum smear-positive vs. smear-negative TB patients.

Biomarker Panel	Sensitivity (95% CI)	
	Smear-positive TB (N = 50)	Smear-negative TB (N = 105)
IFN-γ, IL-6	91.3 (89.5–92.4)	85.7 (84.0–86.0)
IFN-γ, IL-6, MIG	91.7 (89.5–93.3)	85.5 (84.0–86.0)
IFN-γ, IL-6, MIG, IL-18	90.8 (89.5–92.4)	87.5 (84.0–92.0)
IFN-γ, IL-6, MIG, IL-18, CRP	91.5 (89.5–93.3)	88.2 (84.0–92.1)
IFN-γ, IL-6, MIG, IL-18, CRP, GRO	90.8 (89.5–92.4)	88.3 (82.0–94.0)
IFN-γ, IL-6, MIG, IL-18, CRP, GRO, MDC	91.1 (89.5–92.4)	88.1 (82.0–94.0)

Abbreviations: TB, tuberculosis; CI, confidence interval; INF-γ, Interferon gamma; IL-6, Interleukin-6; MIG, Monokine induced by interferon-γ; IL-18, Interleukin-18; CRP, C-reactive protein; MDC, Macrophage-derived chemokine; GRO, Growth-related oncogene.

## Discussion

A simple, low-cost, and accurate TB screening test is a key priority for achieving global TB elimination targets. In this study, we analyzed 44 blood biomarkers and identified at least five (IFN-γ, IL-6, MIG, IL-18, and CRP) that showed potential for utility in TB screening. All five biomarkers had levels that were >1.5-fold higher in TB patients than in patients without TB in the pilot and full datasets, and were the top-ranked biomarkers by variable importance in the full dataset. With sensitivity constrained to ≥90%, specificity was poor—as expected in an inpatient cohort—for all individual biomarkers and for all biomarker panels. However, specificity was higher for biomarker panels than for individual biomarkers, including CRP. Combinations of the biomarkers identified here should be further evaluated among ambulatory PLHIV undergoing systematic screening for active TB.

Our data are consistent with previous studies that have evaluated blood biomarkers in the context of TB diagnosis. Levels of IFN-γ, IL-6, MIG, and IL-18 have all been found to be higher in patients with TB than in healthy controls [21], household contacts [23], or patients undergoing TB evaluation who test negative [24, 26]. CRP has been more extensively evaluated. Sensitivity has been consistently high while specificity has varied with the population being studied (range 6–52% in inpatient studies and 58–81% in ambulatory screening studies [31]). Our study is among the first to evaluate all five of these biomarkers, and the previous studies support that the biomarkers identified here can be expected to have higher specificity in the context of TB screening in ambulatory settings, where patients without TB are less likely to have other illnesses resulting in systemic inflammation.

In addition to the biomarkers identified here, others such as IP-10 have been reported recently to have utility for differentiating patients with and without active TB. For example, Biraro et al found that IP-10 was able to differentiate patients with latent or active TB versus those who were not infected [25] and Mihret et al found that IP-10 plasma levels could differentiate between active TB patients and healthy household contacts [23]. Our study confirmed these findings; however, we replaced IP-10 with MIG for analyses (See Biomarker Selection). Due to the strong performance of IP-10 in our data and prior literature, it should be included along with IFN-γ, IL-6, MIG, IL-18, and CRP in future studies of TB screening in ambulatory populations.

Our study has several strengths, including a rigorous TB reference standard, robust statistical methods, and the use of unstimulated peripheral blood specimens in contrast to many biomarker studies that use stimulated specimens. However, the study also has several limitations. First, the study population was not representative of the majority of patients who would undergo TB screening due to the higher severity of illness and thus greater systemic inflammation in the TB-negative patients in this cohort. However, we successfully identified a smaller number of biomarkers that can be further evaluated in future studies for TB screening in outpatient settings. Second, samples from many patients were not included because TB status could not be clearly defined. Additionally, patients who could not produce sputum were excluded as their TB status could not be classified. However, symptoms and severity of illness were similar among patients included and excluded from the analysis. Nonetheless, the large number of exclusions introduces the potential for bias. Third, the plasma samples used in this analysis had been stored for >2 years and some cytokines/chemokines could have degraded more than others [41]. Last, even if validated in a screening cohort, point-of-care testing platforms would need to be developed before panels of inflammatory markers could be used for TB screening by frontline health workers in high-burden countries.

## Conclusions

Direct measurement of a panel of inflammatory markers, cytokines, and chemokines in peripheral blood is a promising approach to TB screening. Each of the biomarkers identified in this study performed better than symptom-based screening, and performed better than CRP alone when combined into panels. These biomarkers should be further evaluated in outpatient settings where most TB screening occurs and where other illnesses associated with systemic inflammation are less common in order to identify the optimal panel and to determine whether sensitivity and specificity meet targets established by the WHO for a TB screening test.

## Supporting information

S1 Table Median biomarker levels (pg/ml), N = 74. (DOCX)

S2 Table Variable importance of biomarkers, in rank order (N = 74). (DOCX)

S3 Table Diagnostic accuracy of individual top-ranked biomarkers with sensitivity constrained to ≥90% (N = 262). (DOCX)

S1 File Initial and repeat values of cytokines with mean, standard deviation, and coefficient of variation. (XLS)
