# Supplementary material for: Diagnostic performance of blood inflammatory markers for tuberculosis screening in people living with HIV
Source: PLoS One. 2018 Oct 23;13(10):e0206119. doi: 10.1371/journal.pone.0206119 (PMC6198956; doi:10.1371/journal.pone.0206119)
Supplement: S1 Table — (DOCX) [file pone.0206119.s001.docx]

**Supporting Information**

**S1 Table: Median biomarker levels (pg/ml), N=74.**

| **Biomarker** | **No TB (N=25)** | **TB (N=49)** | **Fold Change** | **p-value** |
| --- | --- | --- | --- | --- |
|  | **Median (IQR)** | **Median (IQR)** |  |  |
| INF-γ | 16 (11 - 23) | 45 (24 - 98) | 2.8 | **0.000** |
| MIG | 2713 (1957 - 8362) | 7241 (3466 - 16719) | 2.7 | **0.017** |
| IL-6 | 22 (10 - 41) | 50 (31 - 110) | 2.3 | **0.004** |
| IL-18 | 359 (230 - 465) | 709 (466 - 1255) | 2.0 | **0.000** |
| G-CSF | 103 (79 - 444) | 202 (103 - 349) | 2.0 | 0.258 |
| CRP | 91 (33 - 165) | 169 (120 - 240) | 1.9 | **0.003** |
| IL-8 | 32 (18 - 71) | 56 (30 - 134) | 1.8 | **0.008** |
| IP-10 | 2579 (1767 - 4533) | 4491 (2657 - 7674) | 1.7 | **0.006** |
| TNF-α | 24 (16 - 36) | 36 (24 - 49) | 1.5 | **0.044** |
| SCD-40L | 6522 (3984 - 14929) | 9722 (5814 - 13467) | 1.5 | 0.973 |
| GRO | 1341 (1143 - 2200) | 1981 (1446 - 2616) | 1.5 | 0.086 |
| IL-1α | 20 (10 - 37) | 29 (16 - 42) | 1.5 | 0.112 |
| MCP1 | 386 (203 - 597) | 519 (308 - 897) | 1.3 | 0.129 |
| IL-15 | 7 (5 - 8) | 9 (5 - 15) | 1.3 | **0.040** |
| PDGF-BB | 6492 (3088 - 9189) | 7948 (4909 - 10372) | 1.2 | 0.273 |
| MIP-1α | 11 (6 - 16) | 13 (4 - 25) | 1.2 | 0.719 |
| PDGF-AA | 4095 (2870 - 5990) | 4769 (2517 - 7221) | 1.2 | 0.919 |
| FLT-3L | 25 (11 - 38) | 29 (15 - 42) | 1.2 | 0.496 |
| MCP3 | 20 (8 - 26) | 23 (8 - 32) | 1.2 | 0.311 |
| Eotaxin | 62 (39 - 99) | 69 (54 - 85) | 1.1 | 0.758 |
| VEGF | 218 (148 - 398) | 236 (128 - 349) | 1.1 | 0.771 |
| IL-1ra | 82 (57 - 106) | 85 (53 - 104) | 1.0 | 0.932 |
| IL-5 | 3 (2 - 3) | 3 (1 - 3) | 1.0 | 0.194 |
| TNF-β | 3 (3 - 10) | 3 (3 - 4) | 1.0 | 0.110 |
| IL-13 | 3 (3 - 4) | 3 (2 - 3) | 1.0 | **0.038** |
| IL-4 | 1 (1 - 6) | 1 (1 - 1) | 1.0 | **0.028** |
| GM-CSF | 20 (17 - 25) | 20 (16 - 26) | 1.0 | 0.891 |
| IL-12(p70) | 7 (5 - 13) | 7 (5 - 11) | 1.0 | 0.477 |
| FGF-2 | 56 (41 - 72) | 56 (44 - 84) | 1.0 | 0.806 |
| IL-2 | 3 (2 - 6) | 3 (1 - 5) | 1.0 | 0.695 |
| IL-1β | 3 (2 - 3) | 3 (1 - 3) | 1.0 | 0.445 |
| MIP-1β | 31 (26 - 39) | 31 (25 - 39) | 1.0 | 1.000 |
| IL-3 | 3 (2 - 4) | 3 (2 - 4) | 1.0 | 0.798 |
| IL-9 | 3 (3 - 3) | 3 (3 - 3) | 1.0 | 0.489 |
| Fractalkine | 99 (67 - 126) | 97 (63 - 125) | 1.0 | 0.895 |
| EGF | 48 (31 - 104) | 47 (27 - 93) | 1.0 | 0.648 |
| CCL5 | 2478 (1645 - 3246) | 2165 (1576 - 2559) | 0.9 | 0.345 |
| IL-7 | 13 (10 - 19) | 11 (9 - 14) | 0.8 | 0.110 |
| INF-α2 | 74 (63 - 88) | 61 (40 - 82) | 0.8 | **0.047** |
| MDC | 800 (662 - 1260) | 610 (449 - 862) | 0.8 | **0.011** |
| TGF-α | 4 (3 - 5) | 3 (3 - 4) | 0.8 | 0.063 |
| IL-10 | 18 (9 - 34) | 13 (8 - 35) | 0.7 | 0.701 |
| IL-17 | 7 (5 - 9) | 5 (3 - 8) | 0.7 | 0.057 |
| IL-12(p40) | 37 (22 - 42) | 25 (8 - 43) | 0.7 | 0.212 |
