# Supplementary material for: Diagnostic performance of blood inflammatory markers for tuberculosis screening in people living with HIV
Source: PLoS One. 2018 Oct 23;13(10):e0206119. doi: 10.1371/journal.pone.0206119 (PMC6198956; doi:10.1371/journal.pone.0206119)
Supplement: S2 Table — (DOCX) [file pone.0206119.s002.docx]

**Supporting Information**

**S2 Table: Variable Importance of Biomarkers, in rank order (N=74).**

| **Biomarker** | **Variable Importance** |
| --- | --- |
| INF-γ | 4.7 |
| IL-18 | 4.0 |
| CRP | 2.2 |
| IL-6 | 2.1 |
| MDC | 1.1 |
| GRO | 1.0 |
| IP-10 | 0.9 |
| IL-5 | 0.9 |
| IL-17 | 0.8 |
| IL-8 | 0.7 |
| IL-15 | 0.7 |
| MCP1 | 0.7 |
| PDGF-BB | 0.6 |
| INF-α2 | 0.6 |
| SCD-40L | 0.6 |
| TNF-β | 0.6 |
| Eotaxin | 0.6 |
| MIG | 0.6 |
| IL-13 | 0.5 |
| TNF-α | 0.5 |
| G-CSF | 0.5 |
| IL-1α | 0.5 |
| IL-7 | 0.5 |
| MIP-1α | 0.5 |
| TGF-α | 0.5 |
| Fractalkine | 0.5 |
| PDGF-AA | 0.5 |
| IL-4 | 0.5 |
| CCL5 | 0.4 |
| IL-10 | 0.4 |
| IL-12(p40) | 0.4 |
| VEGF | 0.3 |
| FLT-3L | 0.3 |
| GM-CSF | 0.3 |
| EGF | 0.3 |
| IL-12(p70) | 0.3 |
| FGF-2 | 0.2 |
| IL-2 | 0.2 |
| IL-1β | 0.2 |
| MIP-1β | 0.2 |
| IL-1ra | 0.2 |
| MCP3 | 0.2 |
| IL-3 | 0.2 |
| IL-9 | 0.1 |
