# Supplementary material for: Diagnostic performance of blood inflammatory markers for tuberculosis screening in people living with HIV
Source: PLoS One. 2018 Oct 23;13(10):e0206119. doi: 10.1371/journal.pone.0206119 (PMC6198956; doi:10.1371/journal.pone.0206119)
Supplement: S3 Table — (DOCX) [file pone.0206119.s003.docx]

**Supporting Information**

**S3 Table: Diagnostic accuracy of individual top-ranked biomarkers with sensitivity constrained to ≥90% (N=262).**

| **Biomarker** | **Specificity (95% CI)** |
| --- | --- |
| IFN-γ | 26.8 (22.4 - 31.8) |
| IL-6 | 44.0 (37.4 - 49.5) |
| MIG | 16.3 (11.2 - 20.6) |
| IL-18 | 13.3 (8.4 - 18.7) |
| CRP | 33.1 (29.9 - 36.4) |
| GRO | 1.3 (0.0 - 2.8) |
| MDC | 1.9 (0.0 - 3.7) |

Abbreviations: TB, tuberculosis; CI, confidence interval; INF-γ, Interferon gamma; IL-6, Interleukin-6; MIG, Monokine induced by interferon-γ; IL-18, Interleukin-18; CRP, C-reactive protein; MDC, Macrophage-derived chemokine; GRO, Growth-related oncogene.
